# Multiple antibody specificities (gp41, V1V2, and V3) elicited in the phase II multiclade (A, B, C) HIV-1 DNA prime, rAd5 boost vaccine trial

**DOI:** 10.1186/1742-4690-9-S2-O55

**Published:** 2012-09-13

**Authors:** WB Williams, K Jones, A Krambrink, D Grove, P Liu, NL Yates, MA Moody, G Ferrari, J Pollara, Z Moodie, CA Morgan, H Liao, DC Montefiori, C Ochsenbauer, J Kappes, S Hammer, J Mascola, R Koup, L Corey, G Nabel, P Gilbert, G Churchyard, M Keefer, BS Graham, BF Haynes, GD Tomaras

**Affiliations:** 1 Duke Human Vaccine Institute, Duke University Medical Center, Durham, NC, USA; 2 SCHARP, Fred Hutchinson Cancer Research Center, Seattle, WA, USA; 3 Department of Medicine, University of Alabama at Birmingham, Birmingham, AL, USA; 4 Columbia University Medical Center, New York, NY, USA; 5 Vaccine Research Center, National Institute of Allergy and Infectious , Bethesda, MD, USA; 6 University of Washington, Seattle, WA, USA; 7 The Aurum Institute, Johannesburg, South Africa; 8 Division of Infectious Disease, University of Rochester Medical Center, Rochester, NY, USA

## Background

The phase II DNA prime, rAd5 boost vaccine (HVTN 204) exhibited sufficient safety and immunogenicity to advance into a phase IIb efficacy trial (HVTN 505) in Ad5 seronegative volunteers in the US. In the RV144 ALVAC prime, A/E gp120 protein boost trial, levels of V1V2 IgG antibodies significantly correlated with decreased risk of infection.

## Methods

Sera from 203 vaccinees receiving VRC-HIVDNA016-00-VP DNA (m 0,1,2), and VRC-HIVADV014-00-VP rAd5 (m 6), at two weeks post rAd5 boost, and 208 placebos were examined for binding gp140 and gp120 recombinant proteins, gp41, and an antigen panel of 16 V1V2 IgG scaffolds representing clades A, B and C V1V2 sequences. A subset of vaccinees and placebo was screened for binding CD4BS antigens. Monoclonal antibodies were generated from antigen specific memory B cell sorts and tested for binding, neutralization, ADCC and virion capture.

## Results

Clade A and B V1V2 IgG antibodies were elicited in 38.4% and 19.2% of vaccinees, respectively. A clonal lineage of 3 gp41 mAbs (CH69, CH70, CH71), and a V3-specific gp120 mAb (CH73) were generated from vaccinees. The gp41 mAbs captured infectious HIV-1 transmitted/founder viruses, while CH73 mediated ADCC activity against subtypes B and C infected cells, neutralized subtypes B and C tier 1A viruses, and bound multiple Envs of subtypes A, B and C.

## Conclusion

The phase II multi-clade DNA prime, rAd5 boost vaccine regimen elicited antibody responses to multiple epitope specificities, including V1V2, V3, and gp41. CH69-CH71 and CH73 mAbs represent the initial human mAbs from HVTN 204 vaccine recipients, and reflect the functional profile of vaccine-elicited antibodies. These data suggest that the HVTN 505 Phase IIb efficacy study using this same vaccine regimen may provide an opportunity to examine a diversity of antibody specificities that have been hypothesized as a correlate of HIV-1 infection risk.

